# Detecting Anomalies in Daily Activity Routines of Older Persons in Single Resident Smart Homes: Proof-of-Concept Study

**DOI:** 10.2196/28260

**Published:** 2022-04-11

**Authors:** Zahraa Khais Shahid, Saguna Saguna, Christer Åhlund

**Affiliations:** 1 Division of Computer Science Department of Computer Science, Electrical and Space Engineering Luleå University of Technology Skellefteå Sweden; 2 Information Technology Department Skellefteå Municipality Skellefteå Sweden

**Keywords:** Activities of daily living, smart homes, elderly care, anomaly detection, IoT devices, smart device, elderly, sensors, digital sensors, Internet of things

## Abstract

**Background:**

One of the main challenges of health monitoring systems is the support of older persons in living independently in their homes and with relatives. Smart homes equipped with internet of things devices can allow older persons to live longer in their homes. Previous surveys used to identify sensor-based data sets in human activity recognition systems have been limited by the use of public data set characteristics, data collected in a controlled environment, and a limited number of older participants.

**Objective:**

The objective of our study is to build a model that can learn the daily routines of older persons, detect deviations in daily living behavior, and notify these anomalies in near real-time to relatives.

**Methods:**

We extracted features from large-scale sensor data by calculating the time duration and frequency of visits. Anomalies were detected using a parametric statistical approach, unusually short or long durations being detected by estimating the mean (μ) and standard deviation (σ) over hourly time windows (80 to 355 days) for different apartments. The confidence level is at least 75% of the tested values within two (σ) from the mean. An anomaly was triggered where the actual duration was outside the limits of 2 standard deviations (μ−2σ, μ+2σ), activity nonoccurrence, or absence of activity.

**Results:**

The patterns detected from sensor data matched the routines self-reported by users. Our system observed approximately 1000 meals and bathroom activities and notifications sent to 9 apartments between July and August 2020. A service evaluation of received notifications showed a positive user experience, an average score of 4 being received on a 1 to 5 Likert-like scale. One was poor, two fair, three good, four very good, and five excellent. Our approach considered more than 75% of the observed meal activities were normal. This figure, in reality, was 93%, normal observed meal activities of all participants falling within 2 standard deviations of the mean.

**Conclusions:**

In this research, we developed, implemented, and evaluated a real-time monitoring system of older participants in an uncontrolled environment, with off-the-shelf sensors and internet of things devices being used in the homes of older persons. We also developed an SMS-based notification service and conducted user evaluations. This service acts as an extension of the health/social care services operated by the municipality of Skellefteå provided to older persons and relatives.

## Introduction

Emerging technologies have, in recent years, given rise to the internet of things (IoT). IoT is a combination of smart devices, sensors, and actuators used to connect and interact through the internet and to collect, share and analyze data. Kevin Ashton first coined the term IoT in 1999 to promote radio frequency identification technology [1]. IoT usability and its real-time monitoring capabilities have paved the way for a new range of applications. IoT applications in environments such as smart homes [2], for example, have the potential to support and assist older persons and help them live independently in their homes [3]. These systems can also help indicate the ability of older adults to perform basic daily routines such as cooking and bathing [4].

The need for assistive technologies is driven by the older population, with Sweden's older population, for example, expected to increase by 45% and 87% by 2050 for the age groups of 65-79 years and ≥80 years, respectively [5]. Smart homes fitted with IoT devices can allow older persons to live independently in their homes and allow the “elderly to age in place for twice as long” [6]. Affordable and low-maintenance IoT-based monitoring systems can also provide significant benefits in challenging times, such as the recent global COVID pandemic, particularly for older persons who were the most vulnerable group in the COVID-19 pandemic. A significant proportion of COVID-19 related deaths (48.9%) were among care home residents (the Swedish Public Health Agency [7]). Activities of daily living (ADL) in ambient assisted living applications can therefore play an even greater role in a pandemic through protecting older persons and reducing the pressure on health care providers.

Human activity recognition (HAR) is one of the most important research topics within ADL applications for smart homes. This is at least partially due to the level of support it can provide to older persons and to health care providers. HAR is a challenging and well-researched topic. Advanced IoT devices and low-cost sensors can, however, make activity data collection less expensive [8]. HAR activities can be classified by their granularity and atomic events [9] (eg, open a door). HAR can also help infer high-level activities such as kitchen or breakfast activities through considering contextual environment information [10-15], which only requires a limited number of sensors. The detection of abnormal behaviors by ADL applications is very relevant in health care monitoring systems, particularly in health care systems of older persons where abnormal behavior detection can be of crucial importance [16].

Our research objectives are focused on building a model to identify and learn behavioral patterns and, through this, allow the detection of anomalies in the behavior of older persons using ADL applications and IoT data. This also allows family members to be notified in near real-time. The contributions of our paper are as follows:

- We propose, develop, and evaluate an iVO data analytics architecture for anomaly detection to detect normal and abnormal patterns in the daily activities of older persons.
- We build a statistical real-time anomaly detection method that includes online data processing. 
- We ran our trial in a real-life environment for approximately 64 days for nine different participants, median age 89 years, living in single-resident apartments. We collected data for analysis for approximately 2 years for each household to model the behavior.
- We developed an SMS-based notification service to interact with the relatives of older participants, and we conducted user evaluations. Notifications of normal daily activities and anomalies were sent via SMS to relatives as positive and negative notifications via our developed real-time online system.

The “Internet of Things (IoT) within health and care” (iVO) project [17] started in 2018 and was founded with a focus on older persons living independently in smart home environments. Participant apartments were at three locations in Sweden, in the municipalities of Skellefteå, Kiruna, and Uppsala. Pilot study participants were from Skellefteå municipality, 12 apartments being included, and around 1000 activities being observed over 2 months in the summer of 2020. In the following sections, we present the implementation of this study, the results, discussion, and conclusions.

## Methods

This section describes our research method. This includes the experimental setup, iVO architecture for anomaly detection service, the identification of participant routines and needs (based on interviews), and types of installed sensors.

### Experimental Setup

The experimental study reported in this paper, however, includes 12 participants with a median age of 89 years. Three participants were later excluded due to unexpected life events. We used a statistical method to classify participant behavior into normal and abnormal (anomalies). This was based on whether the amount of time spent on an activity in each room was low or high in duration. The experimental setup worked according to routines collected during the interviews. The main focus was the meal activities.

### iVO Architecture for Anomaly Detection Service

**Figure 1:**
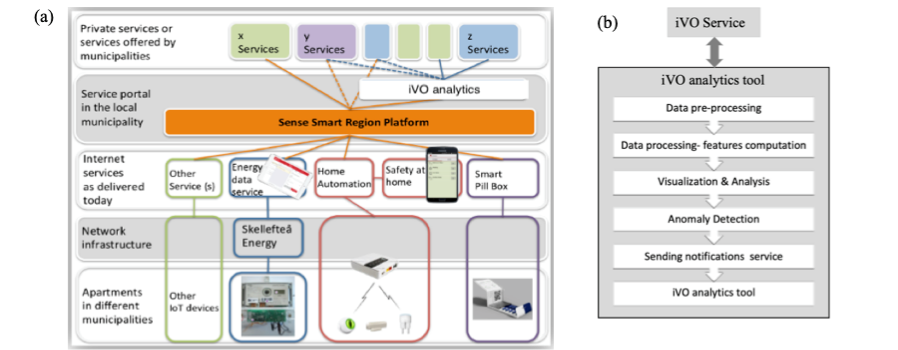
(a) iVO architecture with SSiO platform, (b) iVO analytics. iVO: internet of things within health and care; SSiO: societal development through secure IoT and open data.

The iVO architecture is a layered architecture with horizontal connectivity of different sensors and service providers. iVO architecture is built using FIWARE [18], connecting off-the-shelf sensors and IoT devices via an IoT platform. The platform is called societal development through secure IoT and open data (SSiO) [19]. The SSiO platform was designed and implemented for different IoT applications and services within a smart city, a detailed description of the iVO architecture being given in Saguna et al [20]. The installed sensors are connected via gateways to a service provider (an iVO project partner) to push the sensor data into the SSiO platform, as shown in Figure 1a. The iVO analytics component is shown in Figure 1b.

### Participant’ Routines and Needs Identification Based on Interviews

The project’s first pilot study for the selected age group has been described by Saguna et al [20]. We identified targeted activities through the interview process. The interviews also provided information on the needs of participants and their relatives and activities of interest. The interviews were conducted with the participants along with their relatives who answered questions on the activities they would like the model to recognize, their general opinion of home monitoring, their expectations of implementation, and details about their daily routines. All interviews were documented and recorded by researchers. All participants gave their written consent to use their data.

Most of the participants and their relatives expressed their interest in monitoring meal activities in the kitchen. A matrix of the activities of each participant was constructed based on the environmental setups in each household, on the number of sensors, available kitchen appliances, and the interview responses. The interviewees expressed an interest in receiving both negative and positive notifications. Negative notifications notify that anomalies have been detected in behavioral patterns and positive notifications notify that the activity behavior is normal. This is shown in Table 1.

**Table 1:** End- users' preference for activity recognition.

Apartment ID and Age^a^	Activities^b^	Number of sensors^c^	Notifications
1; 96	Breakfast: 6:00-9:00 Lunch: 11:00-13:00 Bathroom: 22:00-5:00 Bathroom: 00:00-23:59	Total: 9 Water boiler, kitchen & bathroom motion sensor	N/P^d^
2; 91	Breakfast: 7:00-9:00	Total: 11 Kitchen motion sensor, micro	N^e^
3; 94	Breakfast: 7:00-10:00	Total: 8 Coffee machine, kitchen motion sensor	N
4; 99	Breakfast: 6:00-7:00 Lunch: 11:00-13:00 Dinner: 16:00-18:00	Total: 9 Coffee machine, micro, kitchen motion sensor	N
5; 89	Breakfast: 5:00-8:00	Total: 9 Coffee machine, kitchen motion sensor	N
6; 83	Breakfast: 7:00-10:00	Total: 10 Water boiler, kitchen motion sensor	N/P
7; 94	Bathroom: 4:00-6:00	Total: 11 Bathroom motion sensor, water meter	N/P

^a^Apartment ID and age are defined for each participant in this study; each apartment has a single resident.

^b^Meals activities in the kitchen and visitation to the bathroom: from the interview data, we identified the most common routines among all participants, including a start time and end time of each activity.

^c^Number of sensors: the total number of installed sensors at each apartment and the type of sensors used to monitor each corresponded activity in this study implementation.

^d^N/P: negative and positive notifications. The type of notifications that the relatives are interested in receiving on each individual’s activities. Negative and positive notifications represent abnormal and normal behavior in performing the activity, respectively.

^e^N: negative notifications.

### Ethics Approval

The ethical principles raised by and applied to the project were considered in collaboration with the department of homecare at Skellefteå municipality and were approved by the regional ethical committee. The participants gave their consent for the use of their data and the installation of in-home sensors. The project was, overall, in compliance with the European Union’s General Data Protection Regulation guidelines [21]. The data collection and processing included in this study were approved by the Regional Ethical Board in Umea, Sweden (diary no. 2018-189/31).

### Sensors, Data Sets, and Data Preprocessing Module

**Figure 2:**
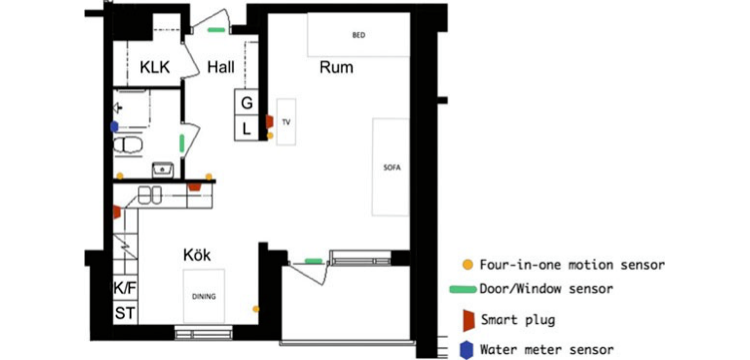
The generic layout of a participant apartment and sensor placements [20].

iVO smart homes use a wide range of off-the-shelf IoT devices and sensors, these systems also being referred to as dense-sensing network technologies [22]. This study, however, only looked at motion sensors, wall plugs, and smart water meters. The sensor installation and floor plan of an older person’s home are shown in Figure 2. All nine apartments have a similar floor plan. Data cleaning is an essential part of the first phase of the study implementation. It is unavoidable that sensors will fail, sensor readings will be lost, and sensor data sets are duplicated, leading to vagueness and imprecision, false positives, and false alarms [23]. This is of even greater importance in older person care health monitoring systems [24]. A reliable monitoring system, therefore, needs to be built before the feature engineering phase is begun. Data cleaning applies, in particular, to motion sensors, redundant data being removed, and missing reading values being identified. Outliers due to visitors or home care visits were excluded.

### Data Processing Module

In this module, we calculated daily duration and visit frequencies for all rooms to extract patterns from and analyze user behaviors. Daily durations are further processed into hourly-based durations. Our analysis used historical data sets from the 2-year data sets. Scalability and re-usability are, however, notable challenges to building individualized activity models [25]. Features engineering is the first step in this module, the time duration feature being the length of time the person spent moving or not moving in the room until transiting to another room/location. The frequency of visits is the number of times the person transits to another room and returns. We used a fixed time window, which is specified by the start and end times of the routines described in the interviews. These time windows, described in Table 1, are used to classify data into normal and abnormal activities.

### Proposed Anomaly Detection Module

Hawkins has defined an outlier as “an observation that deviates so much from other observations as to arouse suspicions that it was generated by a different mechanism” [26]. Detecting anomalies in data has been studied in the statistics community since the 19th century [27]. There are different approaches to detecting anomalies, including the mining-based approach, the logic-based (rule-based) approach, the ontology-based approach [28], and the statistical-based approach [16]. A statistical parametric model can be a simple approach to anomaly detection problems, assuming that the data is normally distributed, fits certain distributions, and that the value of these parameters is unknown. These must be estimated from the given data. Selecting the correct statistical tool for anomaly detection, however, requires the validation of a normality assumption [29].

Normally distributed data can often be tested using histograms. A histogram may not, however, reveal the shape of the distribution. The selection of the normality test tool was based on a comparative study of different normality tests. This study showed that the Shapiro-Wilk test was the most suitable tool for data sets of sample sizes of between 50 and 2000 [30], the null hypothesis in this test being that the sample is a normal distribution. This hypothesis is rejected if P values are less than .05 (95% CI). Results of the tests can be found in Table 2.

**Table 2:** Shapiro-Wilk normality test.

Apartment and activities^a^			Shapiro-Wilk normality test (test statistics *W* and P values)^b^
**1**			
	Breakfast: 6:00-9:00	*W*=.981, *P<*.001 (reject H0)	
	Lunch:11:00-13:00	*W*=.972, *P<*.001 (reject H0)	
2	Breakfast: 7:00-9:00	*W*=.954, *P<*.001 (reject H0)	
3	Breakfast: 7:00-10	*W*=0.926, *P<*.001 (reject H0)	
**4**			
	Breakfast: 6:00-7:00	*W*=.979, P=.047 (reject H0)	
	Lunch:11:00-13:00	*W*=.949, *P<*.001 (reject H0)	
	Dinner:16:00-18:00	*W*=.962, P=.001 (reject H0)	
5	Breakfast: 5:00-8:00	*W*=.967, P=.003 (reject H0)	
6	Breakfast: 7:00-10:00	*W*=.958, P=.009 (reject H0)	
**8**			
	Breakfast: 7:00-10:00	*W* =.943, *P<*.001 (reject H0)	
	Lunch: 10:00-13:00	*W*=.967, P=.005 (reject H0)	
	Dinner: 17:00-20:00	*W*=.729, *P<*.001 (reject H0)	
9	Breakfast: 08:00-10:00	*W*=.982, P=.05 (fail to reject H0)	

^a^Meals activities in the kitchen and visitation to the bathroom: from the interview data, we identified the most common routines among all participants, including a start time and end time of each activity.

^b^If the P value of the Shapiro test is smaller than .05 (the threshold), then the data significantly deviates from a normal distribution.

The normality test results show that 12 out of 13 (92%) tested meal activities in our study do not show a normal distribution. We, therefore, selected Chebyshev’s inequality theorem, a nonparametric statistical method, to detect anomalies [31] in user daily activities based on time duration. Chebyshev’s inequality constructs the upper and lower interval for the percentage of the data that falls outside of k standard deviations from the mean. It holds no assumptions about the distribution of the data and can be used in situations where at least 75% of the data is within 2 standard deviations of the mean. This can be more than 75% in some cases.

The inequality in Equation (1) calculates an upper bound ¼ for the probability of random values exceeding (k) 2 standard deviations from the mean. We, therefore, define an outlier as a data point of a time duration in minutes or hours that exceeds the expected duration by 2 standard deviations [13].

P(|X-μ| ≥ kσ) ≤ 1/k^2^**(1)**

X is the random variable, μ is the mean, and σ is the standard deviation.

A value for p, the significance level of the intervals, is 0.25. This determines which data are potential outliers. To find k using Equation (2):

k = 1/√p **(2)**

The significance level is 0.25 with a confidence level of 75% and a k value of 2. Equation (3) can be used to determine the probability that a randomly selected value is in the interval, around 75% of observations falling within 2 standard deviations of the mean.

μ ± kσ **(3)**

Durations that lie between the lower and upper thresholds are considered to be normal. Data from wall plug sensors was also processed and added to the notifications as additional context. We, however, restricted the model to the duration feature, wall plug sensors, and visit frequencies not being used in the anomaly detection classification process. Our observations showed duration to be the most important feature, duration showing how long a participant remained in a room to perform an activity.

Types of anomalies with example situations:

- Unusually long/short activity: duration in a room in a specific timeframe is unusually long/short, indicating a fall/unconsciousness or health issues [11,15,16].
- Not present: when a user is expected to be in a room in a specific timeframe but is not [13].

### Notification Service Module

**Figure 3:**
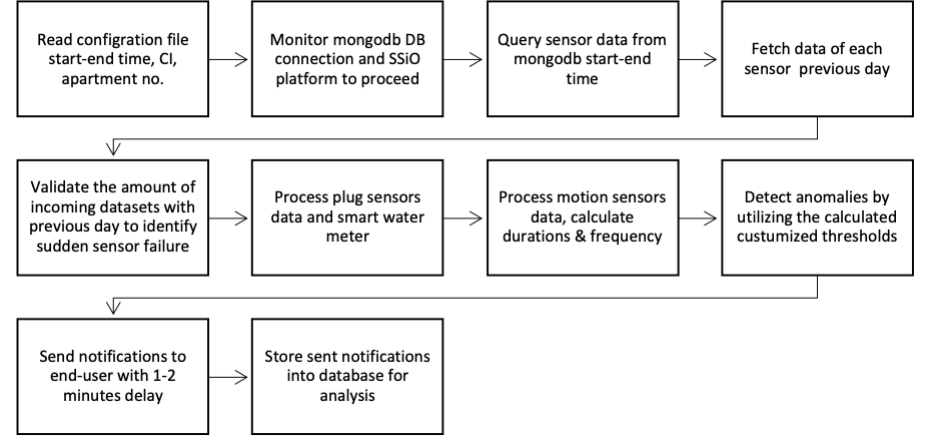
The process for notification service implementation. DB: database; No: Apartment number; SSiO: societal development through secure IoT and open data.

Our implementation delivers positive or negative notifications to relatives and caregivers. Positive notifications represent normal days, and negative notifications represent anomalous days. The notifications were sent via SMS [15]. Negative notifications are sent when activities deviate from the observed normal behavior, which is based on analyzed historical data. Positive notifications are sent when the user’s behavior is normal and when the relative expresses an interest in receiving such notifications. The design of the notification service process in Figure 3 starts by reading a configuration file, which automates a schedule and starts the service process.

We contacted the relatives before running the system to inform them of the activities they would be notified about. They were shown a notification format and the information that would be included. They were optimistic about the possibilities that the system could provide, the system providing comfort through being able to remotely check in on their parents daily. A consensus was reached with participant relatives on the content of the notification and the type of communication to be used (ie, SMS).

The notification structure included the number of minutes spent on the meal activity, across how many visits, whether they used any appliances, and the normal duration of the activity for that participant based on their identified patterns. An example of the structure and information for one positive notification sent for apartment 1 is shown below:

50.4 minutes of activity in the kitchen between 07.00 and 10:04, during 8 visits. The kettle has been used. Our analysis shows that 9-70 minutes is the normal time duration in the kitchen. If that message is not correct and deviates from the actual event, we are grateful for feedback with a description of the deviation.

LTU iVO Message ID: 1, 2020-07-21

The relatives were given the option to reply to every notification. This response included feedback when there was anomalous behavior.

## Results

**Figure 4:**
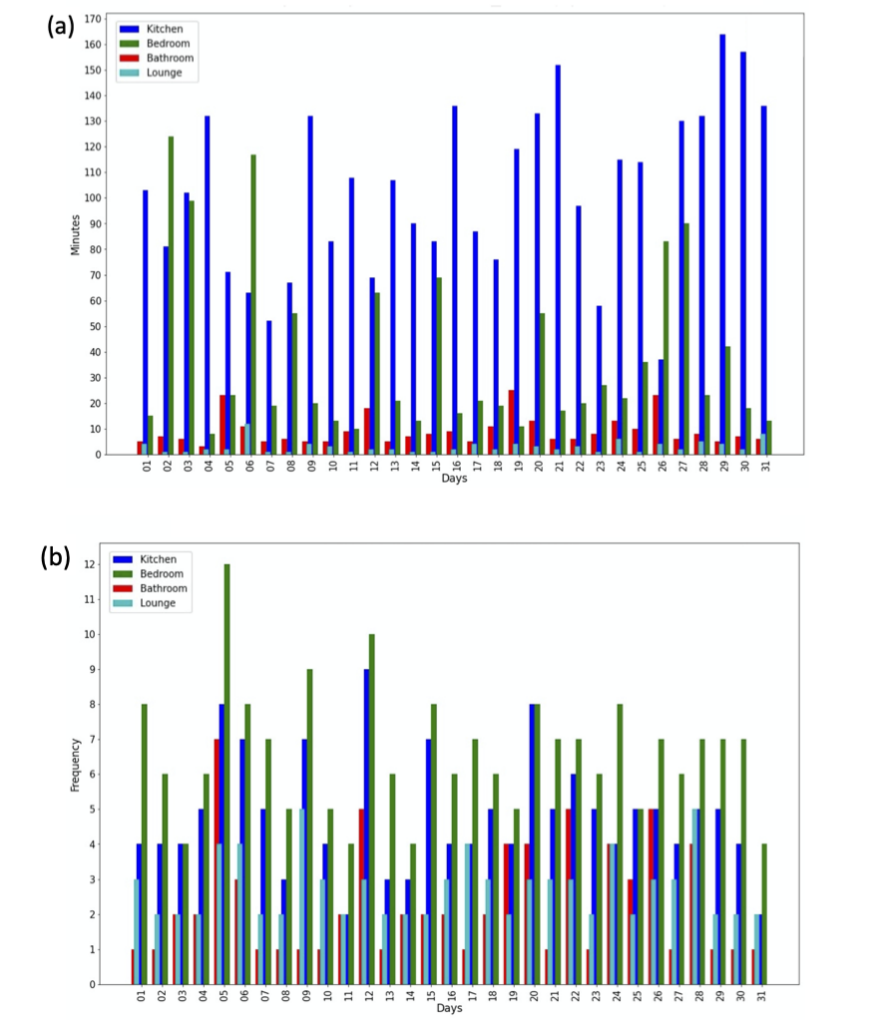
(a) Data for the duration in all rooms between 06.00 and 10.00 in apartment 1 (August 2020), (b) Data for the number of visits to all rooms between 06.00 and 10.00 in apartment 1 (August 2020).

Duration and visit frequency are essential in the analysis of user daily behavior. Figure 4a shows time durations in minutes in different rooms of the apartment, frequencies of visits being shown in Figure 4b. The participant in apartment 1 spent more time in the kitchen than in the other rooms, except for 2 days in the trial month. This indicates that the participant is active during the morning due to eating breakfast. The number of transitions from and to the bedroom in Figure 4b shows that the participant is active, the participant remaining in the bedroom for only short periods of time. Such behavior conforms with a typical morning.

Visits to the bathroom also show consistency, multiple visits for short periods of less than 10 minutes. The duration in the bathroom for four days on the 5th, 12th, 19th, and 26th of August 2020 was, however, 20-25 minutes. These days were cross-checked with this participant’s profile and showed that home care visited the participant to help them with bathing. The participant spent less time in the bedroom and more time in the kitchen on most weekend days (1, 2, 8, 9, 15, 16, 22, 23, 29, 30) in August. The kitchen was the most used room in the apartment, especially in the morning, as opposed to in the lounge and bedroom. User behavior can easily be interpreted from durations and transitions between the rooms.

The value of the inequality is used when we only know probability distribution estimates, mean and standard deviation. These approximations are derived from the historical data sets of extracted features of time duration in the kitchen, as the example in Figure 4a shows.

**Figure 5:**
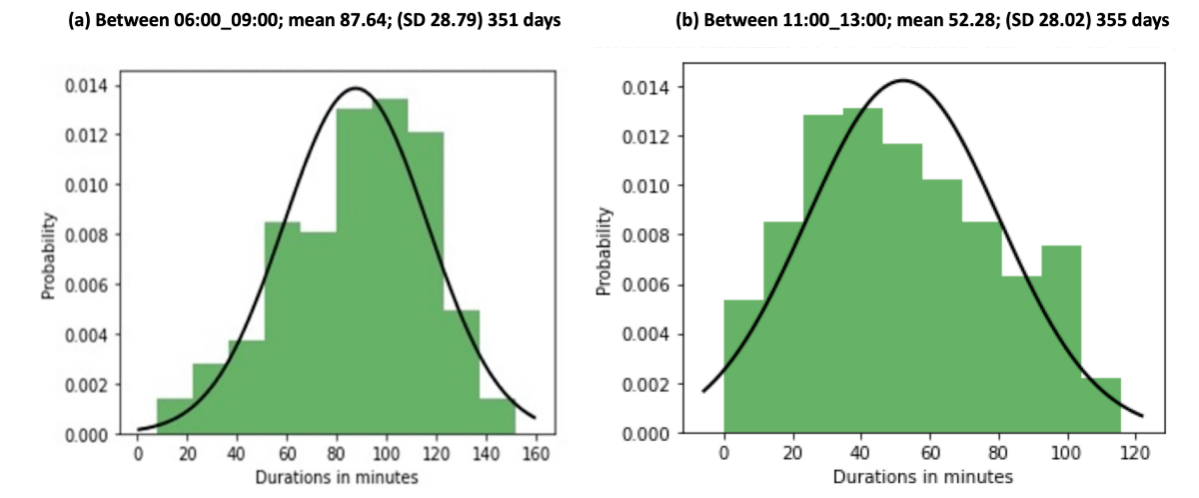
Probability distribution with mean (SD) for 351 days in the kitchen between (a) 6:00 and 9:00 and (b) 11:00 and 13:00 in apartment 1.

Figures 5a and 5b show the distribution of time durations of two-meal activities for 351 days in 2019 and 2020. It can be seen that the participant spent between 1-2 hours in the kitchen in the morning on 250 of the 351days (Figure 5a). There are only a few days in the year when the participant spent less than half an hour and more than two hours in the kitchen during a year, these being on the extreme right and left sides of the distribution.

**Figure 6:**
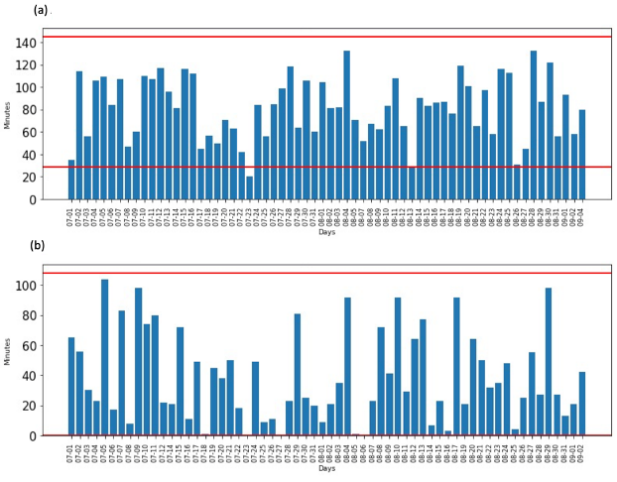
(a) Data of durations in kitchen between 06:00 and 09:00 in apartment 1, (b) Data of duration in kitchen between 11:00 and 13:00 in apartment 1, (red lines represent lower/upper bounds) for July and August 2020.

Figures 6a, Figures 6b, and Multimedia Appendices 1, 2, and 3 show the activity of a participant in the kitchen in the morning and mid-day. Figure 6a shows the time spent in minutes in the kitchen between 6:00 and 9:00 in July and August 2020. The red lines represent the calculated minimum and maximum thresholds and are based on the mean and standard deviation information given in Figure 5, which was calculated from the 2019 and half of 2020 data (355 days). This provides valuable information on probability.

About 80% of the duration lies within the range of 29 to 145 minutes. The kitchen was not used by the participant in the breakfast activity time range on August 6 and September 3, this being anomalous. The time duration for lunch activity between 11 and 13 (Figure 6b) shows either very short or long stays. It is noticeable that the level of activity is low in the kitchen, which could be due to lunch only being prepared in the kitchen but eaten in another room.

Table 3 summarizes the estimates of each average mealtime duration as calculated from the historical data (80 to 355 days) for eight apartments. The values are depicted in Figure 5 and Multimedia Appendix 4. The number of trial days of each observed mealtime and the average duration in the notification trial between July and August 2020 is also depicted.

**Table 3:** Average duration in the kitchen as observed from total historical days is consistent with average duration in the kitchen as observed from total trial days.

Household and mealtime from interviews^a^		Total historical days^b^	Average Duration for historical days^c^	Number of trial days^d^	Average Duration for trial days^e^
**Apartment 1**					
	6:00-9:00	351	87	69	82
	11:00-13:00	355	52	66	39
Apartment 2	7:00-9:00	364	34	51	32
Apartment 3	7:00-10:00	144	40	60	42
**Apartment 4**					
	6:00-7:00	126	24	56	23
	11:00-13:00	140	47	42	63
	16:00-18:00	137	44	46	38
Apartment 5	5:00-8:00	128	29	46	30
Apartment 6	7:00-10:00	81	41	52	41
**Apartment 8**					
	7:00-10:00	115	40	62	41
	10:00-13:00	119	41	64	44
	17:00-20:00	115	17	57	23
Apartment 9	8:00-10:00	145	55	45	73

^a^Meals routines in the kitchen collected from the interview data, including a start time and end time for each.

^b^Number of past days defines regular activity patterns defined from the records in 2019 and the first half of 2020 data.

^c^Average duration associated with each meal during 2019 and the first half of 2020 data.

^d^Number of observed days during the trial July-August 2020 for each meal activity.

^e^Observations of average duration associated with each meal during the trial July-August 2020.

For example, participant 1 follows a pattern of performing the breakfast activity between 6:00 and 9:00. This shows that she was consistent in her habits. The regular pattern derived from the historical data showed a duration of 87 minutes, while observations from the notification gave a duration of 82 minutes. Most of the participants are consistent in their habits, following a pattern in daily meal activities. The activities also occurred within the self-reported timings.

We observed approximately 1000 meals in nine apartments between July and August 2020. We also observed bathroom activity. Table 4 shows the number of positive and negative notifications for normal and anomalous behavior sent to each household’s relative. The notifications are based on the end-users wishes defined in Table 1.

**Table 4:** Types of notifications sent for eight apartments for each mealtime during July-August 2020.

Apartment ID, type of notification^a^, and total number of observed mealtime activities^b^	Mealtime from interviews^c^	Number of observed activities for each mealtime^d^	No. of normal activity/positive notifications^e^	No. of anomalies/negative notifications^f^
**1: N/P^g^ (n=135)**				
	6:00-9:00	69	56	13
	11:00-13:00	66	63	3
2: N^h^	7:00-9:00	51	49	2
3: N	7:00-10:00	60	58	2
**4: N (n=144)**				
	6:00-7:00	56	56	0
	11:00-13:00	42	40	2
	16:00-18:00	46	46	0
5: N	5:00-8:00	46	46	0
6: N/P	7:00-10:00	52	51	1
**8: N/P (n=183)**				
	7:00-10:00	62	51	11
	10:00-13:00	64	60	4
	17:00-20:00	57	52	5
9: N	8:00-10:00	45	40	5
Total		716	668	48

^a^The type of meals notifications, negative and/or positive, for each apartment received based upon their requests

^b^The total number of observations of each apartment’s meals activities.

^c^Meal routine activities in the kitchen collected from the interview data, including a start time and end time for each.

^d^Number of observed activities per apartment per meal activity, breakfast/lunch/dinner during the trial July-August 2020.

^e^The number of normal activity of users during the trial (July-August 2020) and that match the regular activity patterns defined from the 2019 and first half of 2020 data.

^f^The number of anomalous activity of users during the trial (July-August 2020) and that deviated from regular activity patterns defined from the 2019 and first half of 2020 data.

^g^N/P: negative and positive notifications.

^h^N: negative notifications.

For example, about 80% (56) of our observations of breakfast activity for participant 1 fall between –2 and +2 standard deviations of the mean. Fifty-six (80%) days were normal, and 13 of the 69 observed days (19%) were abnormal. Normal activity falls between the 29 and 145 minutes minimum and maximum threshold, respectively. The majority of meal notifications (119/135, 88%) were sent to participant 1 in the notification trial to confirm her expected morning and lunch meal behavior.

The regular patterns derived from the historical records and the observations in the trial show that the participant’s behavior closely matches the routines they self-reported in the interviews. The participant regularly follows their breakfast and lunch activity routines, including timing. These routines are therefore reliable enough to be used for alerts.

A total of 716 meal activities were observed for the 8 apartments, 668 (93%) being normal and 48 (7%) being abnormal. The participants are within the same age group. It can therefore be noted that approximately 93% of the total 716 meal activity observations for all apartments corresponded to the expected behavior.

Our system sent out 421 notifications to 8 apartments. Some of the apartments expressed an interest in receiving positive and negative notifications. Three hundred seventy-three (88%) positive notifications were sent, corresponding to normal behavior during breakfast, lunch, and dinner; 48 (7%) negative notifications were sent, corresponding to anomalous behavior. More than 75% (537/715) of observed meal activities for all apartments fell within 2 standard deviations of the mean and so correspond to normal behavior. The results closely match the participant routines reported in the interviews.

Feedback on the effectiveness of and user satisfaction with the system was collected. This can provide information on the actual well-being of older persons and can also help relatives to understand that monitoring systems can improve current interventions but cannot substitute the existing health care system. The feedback results from 5 apartments out of 9 are shown in Table 5. The table summarizes the non-mandatory weekly questions sent to end-users on their satisfaction with the notifications received, end-users scaling our service on a scale of 1-5. The results show a positive user experience. We received an average of 4 on a 1-5 Likert-like scale, one being poor, two fair, three good, four very good, and five excellent. The relative of the apartment 6 participant, for example, responded to the notification: “No activity in the kitchen between 05:00 and 07:59.” The feedback was: “I was in contact with my mother at 08.03, and she was in the kitchen..” Another example of feedback was from a relative of the apartment 4 participant, who responded to a change in dinner pattern. Notification: “No activity in the kitchen between 16:00 and 17:59.” Feedback: “Late dinner!” This informed our system of the pattern change and validated our anomaly recognition method. Relatives were also asked to provide weekly feedback via SMS on our system’s overall performance. Weekly notification: “Hello, How have you experienced our iVO. LTU notifications in the last week on a scale of 1-5 (5 is best)? Thank you for taking part!” The participant in apartment 6, for example, in the second week of feedback expressed his satisfaction with the accuracy of notifications. “At the same time, every day, we get a positive notification, which I think is good.” The overall experience was positive, the relatives finding it helpful to know when to contact the participants, based on their normal/abnormal activity routines. The notifications also allowed them to be informed that “all is well” with older participants, which is the overall focus of the iVO project.

**Table 5:** Summary of weekly feedback from relatives of each apartment (ID) on a scale of 1-5 where 1 is poor, and 5 is excellent.

Date	H2	H4	H5	H6	H9
2020-07-08	5	5	3	No-reply	No-reply
2020-07-17	5	5	No-reply	No-reply	No-reply
2020-07-27	5	5	3-4	4	No-reply
2020-08-04	4	5	No-reply	4	No-reply
2020-08-12	4	5	3-4	4	4
2020-08-18	5	5	3-4	No-reply	No-reply

## Discussion

### Principal Findings

Our study demonstrates, through nine cases, how a data-driven approach and longitudinal data from interviews can convert large amounts of sensor data into knowledge. Our approach can detect anomalies in ADLs and utilize notifications to alert relatives of these anomalies. An important aspect of this approach is that it can facilitate the interpretation of data from real-world smart homes and use this in real-time monitoring systems to identify activities that deviate from the normal patterns of older persons. This enhances our understanding of personalized setups and different individual daily routine preferences. The results support the use of data from off-the-shelf sensors and IoT devices (installed in real homes) and the improvement of health care services by feedback to caregivers in near real time. This further enables older persons to live independently in their homes for longer.

### Comparison With Prior Work

We used, in our research, statistical methods to detect anomalies, an approach that has been used in other studies [13,16,32]. The statistical methods we used to detect anomalous behavior in ADLs are in line with previous studies. We, however, in the interests of accurate interpretation, tested the assumptions of data normality. Konios et al [32] recruited 30 volunteers aged 30 to 45 years to conduct a study of the recognition of low-level activities such as steps in preparing and drinking tea/coffee. They used the mean ±1.5 standard deviations as the threshold for classifying normal and anomalous events. Another similar study [16] used low-level activities from the annotated CASAS public data set [33]. The mean and ±1 standard deviation were used to classify activities as normal and abnormal behavior. The mean ±2 standard deviations were, however, used in [13] to detect anomalous behavior at the 95% CI, thresholds being calculated using 2 months of history data of students’ working activity. Our method uses Chebyshev’s inequality, thresholds being based on intervals in which only 75% of the data is within 2 standard deviations, 95% being within 2 standard deviations in a normally distributed data set.

Our results are distinct in that they were collected in an uncontrolled environment and used real-world data of older participants in their homes. Konios et al [32], in contrast, used a lab setting; Jakkula et al [13] used synthetic data and one day of real data from a lab environment to validate their approach. The work in Paudel et al[16] was implemented using annotated public data sets.

Our overall approach builds on earlier work [12,14,34], all being real-world implementations. The data collection process, sensor setup, the method used for pattern identification, and the behavior of older persons, however, differ. Beunk et al [12] aimed to visualize sensor information, duration, and start time from log data, notifications being sent to caregivers, participants, and relatives using real-world monitored activities of 5 participants aged ≥ 65 years. Kasteren et al [14] used 180 days of real-world data obtained from power usage, motion sensors, and interviews to carry out a behavioral analysis of the 3 participants. The distribution of daily activities aggregated over multiple days was visualized using radar plots. Interviews and motion sensors were used to identify residents' sleep and daily movements. Another longitudinal study [34] evaluated the usage of unobtrusive technologies in detecting a change in activities and cognitive decline by statistically analyzing 200 days of data on the daytime and nighttime activities of 233 senior participants with a mean age of 83 years.

Our study proposes a solution that detects anomalies in different types of activities in real-time. Our approach is validated by comparing longitudinal data collected from interviews with observed patterns collected from historical data (80-355 days of sensor data). This was processed to classify activities into normal and anomalous behavior and so allow notifications to be generated.

#### The Strengths of the Used Approach

Routines are defined as being designed behavioral patterns that are used to orchestrate activities. The clock, time duration, contexts, and order are also used in this [35]. Data collected from participants on routines helps validate the analysis of sensor data and so contributes to the minimization of false positives [14]. This also helps more reliable and personalized notifications to be delivered, which helps ensure that the needs of older persons are addressed. Adherence to regular daily routines by older persons contributes to a reduction of stress, increases feelings of safety [36], and improves sleep quality [37]. Our proposed approach to ADL analysis used routine data (data collected from the interviews) of older participants to identify anomalies in ADLs. This approach is similar to that used by other studies [12,14,34]. Our approach, however, differs from [13,16,32], which used annotated activity data. Our study relies on sensor labels and routines defined in the interviews.

HAR activities are, as in Saguna et al [10], classified as low-level activities such as walking and high-level activities such as making coffee. It is important, as mentioned in Hussain et al [38], to use historical sensor data to analyze individual high-level activity behavior patterns (ie, ADLs). Obtaining the required historical data from real-world environments is, however, challenging, especially for older adults. Activity recognition with a focus on health care could also be defined as behavior recognition, this relying on historical data captured from sensors to infer ADLs as high-level activities. This type of high-level activity recognition could reduce human resource costs, allowing the detection of anomalies from the normal behavior of older persons and caregivers to be informed of this.

Some studies [15] collected data from a controlled environment and for nonolder participants [13]. The findings from these are, however, difficult to generalize to the ADL applications of older persons. Most of the research work within HAR [39] also relies on annotated public data sets generated in controlled environments or lab settings [40]. Solutions based on controlled environments may not be suitable for real-world deployments. We, however, collected historical data from motion and wall plug sensors for time periods ranging from 3 months to one year and for nine real homes.

Activity data can be tracked and collected from multiple sensor technologies, wearable devices often being used in activity monitoring systems to capture the data of older persons [22] and to infer low-level activities such as walking or falling [8]. Wearable devices, however, lack practicality, especially for older users [38]. Hernandez et al [40] also investigated previous HAR work between 2014 and 2019 and showed that data is primarily collected from mobile and wearable devices rather than dense-sensing networks.

The deployment of dense-sensing networks in anomaly detection systems is highly recommended in the health care systems of older persons. This is due to their practicality and to their robustness to changes in the environment [22]. These networks can also gather more general information, which can be used to recognize ADLs (ie, high-level activities such as leaving home or sleeping) [8]. In this trial, we used the data from four motion sensors in each apartment to analyze the behavior of users, this being a similar approach to that used in Beunk et al and Kasteren et al [12,14].

The strength of our approach lies in the handling of noise arising from off-the-shelf sensors, which are prone to malfunctioning [41]. This noise is handled by building mechanisms that eliminate erroneous readings or noise from the preprocessing of sensor data. This ensures better accuracy than previous approaches [42] and reduces false alerts [24].

Detecting anomalies in activity patterns using time series sensor data without annotations is challenging. We, however, analyzed the behavior of older persons by developing algorithms that can build temporal features such as duration in and the number of visits to each room. Determining changes in these activities using duration time is, therefore, an important development in the analysis of behavioral patterns [35]. Anomalies can therefore be detected using these temporal features. There is a need for near real-time technology in support of older persons in their activities. Examples of this include reminders to take medicine and interaction to provide immediate support in ongoing activities such as preparing a meal [8,43]. The data processing in real-time ADL analysis is challenging [39]. Our approach, however, uses near real-time analysis in the collection, monitoring, preprocessing, and processing of sensor data for each apartment.

#### Effectiveness of the Developed iVO System

The estimated probability distribution of each activity and for each apartment is given in Figure 5 and Multimedia Appendix 4. The activity patterns are reflected in the mean time duration for each activity, which is drawn from historical data and is given in Table 3. There is a close correspondence between the historical patterns and the test data set patterns for morning activities (notification trial July-August 2020) for all eight participants. The historical data set shows that they use on average 44 to 47 minutes on breakfast/lunch, the trial data showing that they use 45 to 50 minutes. There are, however, some variations, such as the dinner activity of 2 apartments (4 and 8) and the lunch activity of 3 apartments (1, 4, and 9). The average duration for each participant based on the historical data and the notification trial shows routine consistency. The strength of our approach is the ability to identify the regularity in the timing and duration of the different ADLs and to from this identify deviations from this. The importance of the timing, duration, and regularity of activity routines is highlighted in Chung et al [44], and changes in routines, furthermore, potentially signaling cognitive decline or a health issue. Our approach showed that most participants followed a routine in their activities in the trial period.

The most suitable method for constructing thresholds must be selected, the correct handling of changes or irregularities in routines and a reduction in false alerts being dependent on correct selection [14]. These methods need to be reliable if our anomaly notifications are to be effective. We, therefore, set up notification alerts for three types of anomalies: (1) not present, (2) maximum time spent exceeded, and (3) minimum time spent not reached. Our approach used the historical data used to determine statistical thresholds for anomalies (2) and (3), assumptions including data normality, homogeneity of variances, and linearity. These assumptions are important in the selection of a suitable method, their violation leading to result misinterpretation [29]. Previous work has used statistical methods [13,16,32]. We, however, perform the normality test (Table 2), which allows us to determine an appropriate statistical method—Chebyshev’s theorem for classifying normal and anomalous behavior. Identifying changes using this method allowed us to consider confidence levels of 75% to be normal. This was, however, 93% for all participants and all activities.

The number of activities monitored, identified, and notifications generated in the trial period also reflect the effectiveness of our system. Our system observed 716 meal activities in the trial period in 8 apartments, 668 (93%) being normal and 48 (7%) being anomalies. Our system generated 421 notifications, 373 (88%) positive notifications that correspond to normal behavior during breakfast, lunch, and dinner, and 48 (7%) negative corresponding to anomalies.

### Implications

Our findings show that our system can benefit relatives and can be used by formal health care providers in Skellefteå municipality. Relatives and caregivers received timely notifications of older participants’ activities. The system was also able to distinguish between normal and anomalous behavior, which can be used to detect long-term changes in routines and which can signal the early stages of cognitive decline. We believe that this type of system can have a direct impact on the enabling of older persons to live independently for longer. Feedback collected on our system (Table 5) was mainly positive and reflects a high level of user satisfaction with the iVO service. Some participants showed an interest in continuing the service after the trial period ended.

### Limitations

Our approach is limited to the use of duration to identify anomalies. The root cause of the anomalies can, however, only be known via feedback from relatives. A direct method for establishing the root causes of anomalies would help us to understand anomalies better. This approach would, however, make our system too intrusive, interrupt the day-to-day lives of participants, and have an impact on their normal routines. Our preference is that relatives communicate with participants about anomalous behavior and that feedback to our system is then provided by relatives. The use of Chebyshev’s inequality is another limitation. Thresholds are based on loose intervals with only 75% within 2 standard deviations. This can be compared with a normally distributed data set where 95% is within 2 standard deviations. These loose intervals mean the thresholds are wider apart and can therefore lead to fewer anomalies.

### Conclusions

We developed an SMS-based notification service based on information provided in interviews on the needs of participants and their relatives, on the activities they were interested in, and on data from the off-the-shelf sensors and IoT devices installed in homes. We also conducted user evaluations. This service acts as an extension of the municipality health/social care services and helps older persons to live in their homes independently. We proposed, developed, and implemented an anomaly detection framework for the recognition of anomalous daily activities of older persons living in single resident smart homes, using the real-life uncontrolled setting of 9 older participants. This paper proposed a probabilistic approach to the temporal analysis of ADL of nine older participants in a real-world environment. The method introduces a way of indicating whether the probability of a performed activity is considered to be normal or anomalous behavior using duration.

Our system observed approximately 1000 meals and bathroom activities. Notifications were also sent to 9 apartments between July and August 2020. Four hundred twenty-one notifications (59%) out of the observed meals activities (716) were sent to 8 apartments on each meal activity, with our approach considering more than 75% (537) of observed meal activities to be normal. This figure was, however, in reality, 93% of meal activities, these falling within 2 standard deviations of the mean and so corresponding to their normal behavior. The behavior patterns derived from the historical processed sensor data closely match the routines participants reported in the interviews. We received positive user experience feedback on the service from 5 out of 9 participants’ relatives (55%) and an average of 4 points on a 1-5 satisfaction scale. The results ultimately support the use of IoT devices in homes as an extension of health/social care services, which can, in turn, increase the opportunity to age in the home independently.

### Future Work

We will, in the next phase of this project, use advanced data analytics methods to further investigate the data. We would include other types of anomaly classes in this, such as transitions or visits of unusually short durations, which can indicate unrest and the detection of an unusual presence. Other types of context sensors and features also need to be considered in the analysis. Contextual information such as weather conditions and holidays could, for example, improve results and reduce false positives in detected routines [45]. Future research can furthermore test algorithms that monitor real-time ADLs using data for that specific week, which will allow for weekly variations in routines. Data based on a monthly moving window would also allow for seasonal variations in routines. We are working towards using reinforcement learning as a multi-armed bandit problem (MAB). We plan to conduct follow-up interviews for the study as part of a workshop to communicate our results and understand further needs. Direct feedback from older persons is another important aspect. This would, however, increase the cognitive load upon them. There was no appropriate communication channel for feedback on the alerts or activity updates sent to relatives by older persons, which was not implemented due to the increased burden of daily feedback on older persons. This type of feedback was mainly gathered via the relatives, who communicated with their older relatives about an anomalous notification. This will be addressed further in our future research.

## Appendix

Multimedia Appendix 1 Observations of participants in apartments 2-9 for different meal activities in the kitchen during the trial, July and August 2020. The redlines represent the lower and upper bounds.

Multimedia Appendix 2 Observations of participant's visits frequency and water boiler usage during breakfast in the kitchen in apartment 1 during the trial, July and August 2020.

Multimedia Appendix 3 Observations of participant's visits frequency and water boiler usage during lunch in the kitchen in apartment 1 during the trial, July and August 2020.

Multimedia Appendix 4 Probability distribution (PDFs) of the duration of each meal activity of each participant, apartment 2-9, during 2019 and the first half of 2020. The total number of days varies from 80 to 355 days of different apartments, including the mean (mu) and standard deviation (std) duration values of mealtimes.

## Abbreviations

ADL: activity daily living
HAR: human activity recognition
IoT: internet of things
iVO: internet of things within health and care
SSiO: societal development through secure IoT and open data
