# Correction: Detecting Anomalies in Daily Activity Routines of Older Persons in Single Resident Smart Homes: Proof-of-Concept Study

**DOI:** 10.2196/58394

**Published:** 2024-04-30

**Authors:** Zahraa Khais Shahid, Saguna Saguna, Christer Åhlund

**Affiliations:** 1 Division of Computer Science Department of Computer Science, Electrical and Space Engineering Luleå University of Technology Skellefteå Sweden; 2 Information Technology Department Skellefteå Municipality Skellefteå Sweden

In Detecting Anomalies in Daily Activity Routines of Older Persons in Single Resident Smart Homes: Proof-of-Concept Study (JMIR Aging 2022;5(2):e28260), the authors noticed one error.

In the orinally published article, a duplication error occured in Figure 5. The original Figure can be viewed in Multimedia Appendix 1.

Figure 5 has been corrected as follows:

**Figure 5:**
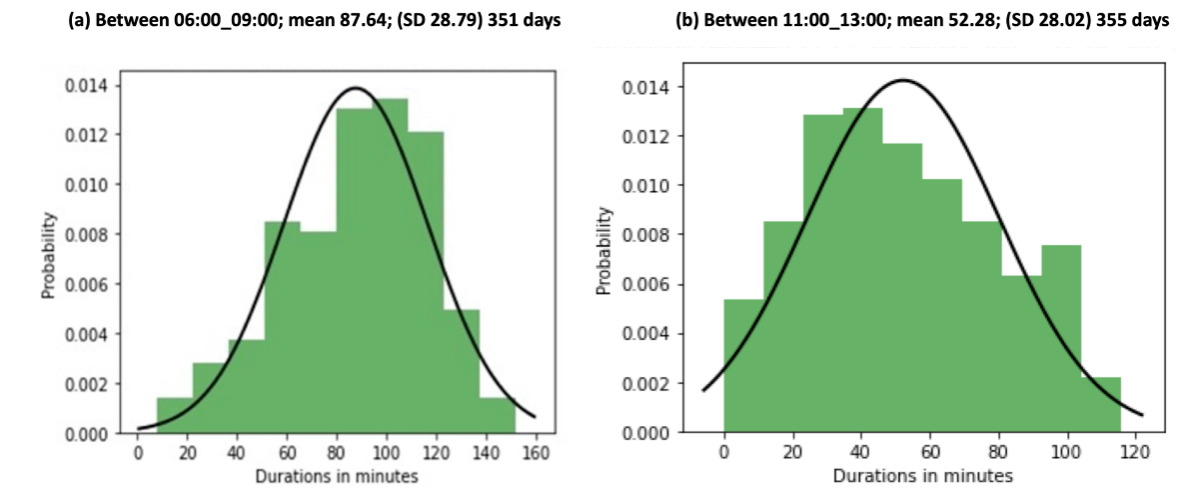
Probability distribution with mean (SD) for 351 days in the kitchen between (a) 6:00 and 9:00 and (b) 11:00 and 13:00 in apartment 1.

The correction will appear in the online version of the paper on the JMIR Publications website on April 30, 2024, together with the publication of this correction notice. Because this was made after submission to PubMed, PubMed Central, and other full-text repositories, the corrected article has also been resubmitted to those repositories.

## Appendix

Multimedia Appendix 1 Originally published Figure 5.

