# Supplementary figures and images for: Detecting Anomalies in Daily Activity Routines of Older Persons in Single Resident Smart Homes: Proof-of-Concept Study
Source: JMIR Aging. 2022 Apr 11;5(2):e28260. doi: 10.2196/28260 (PMC9039812; doi:10.2196/28260)

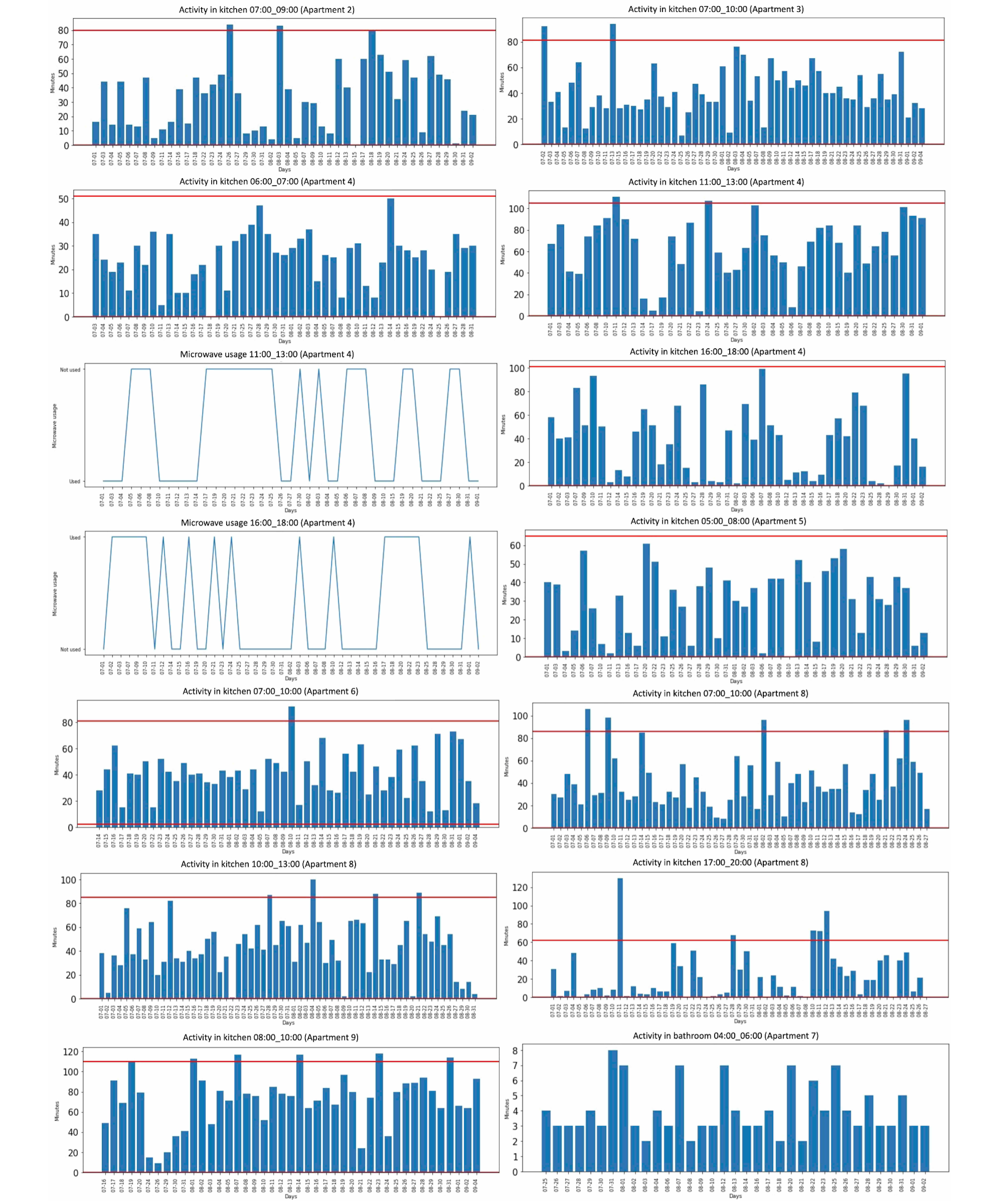

Supplement: Multimedia Appendix 1 [file aging_v5i2e28260_app1.png]

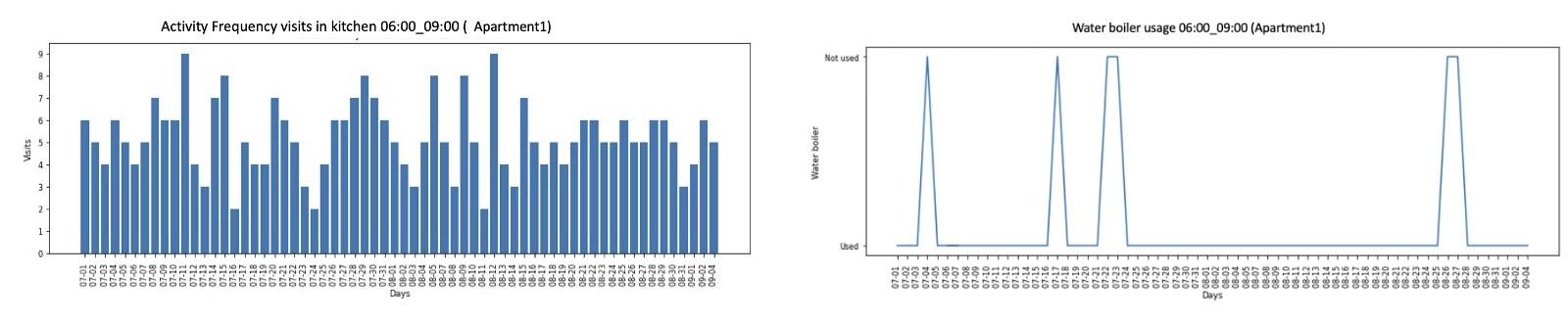

Supplement: Multimedia Appendix 2 [file aging_v5i2e28260_app2.png]

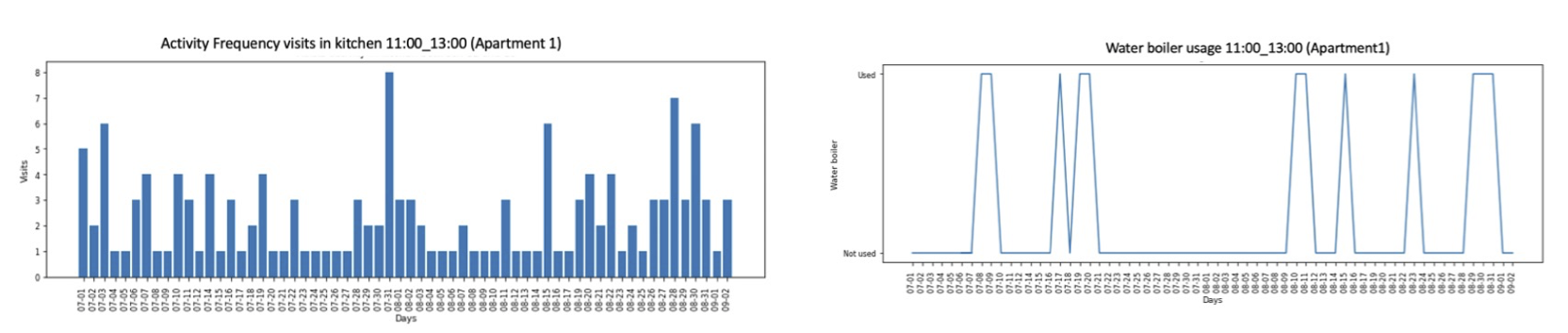

Supplement: Multimedia Appendix 3 [file aging_v5i2e28260_app3.png]

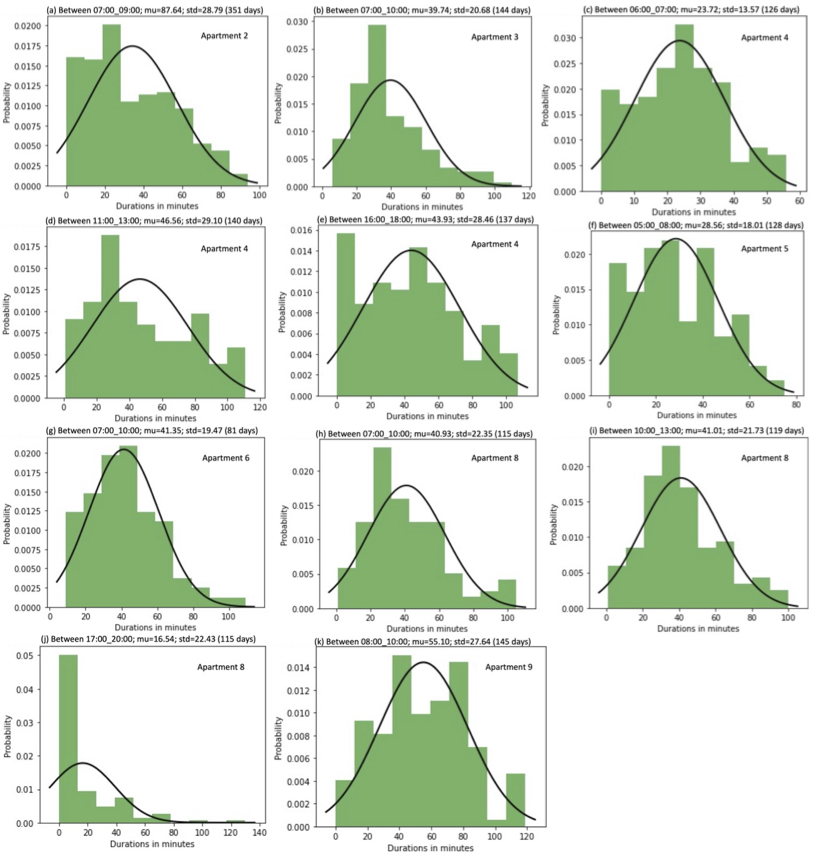

Supplement: Multimedia Appendix 4 [file aging_v5i2e28260_app4.png]
