# Supplementary figures and images for: Correction: Detecting Anomalies in Daily Activity Routines of Older Persons in Single Resident Smart Homes: Proof-of-Concept Study
Source: JMIR Aging. 2024 Apr 30;7:e58394. doi: 10.2196/58394 (PMC11094591; doi:10.2196/58394)

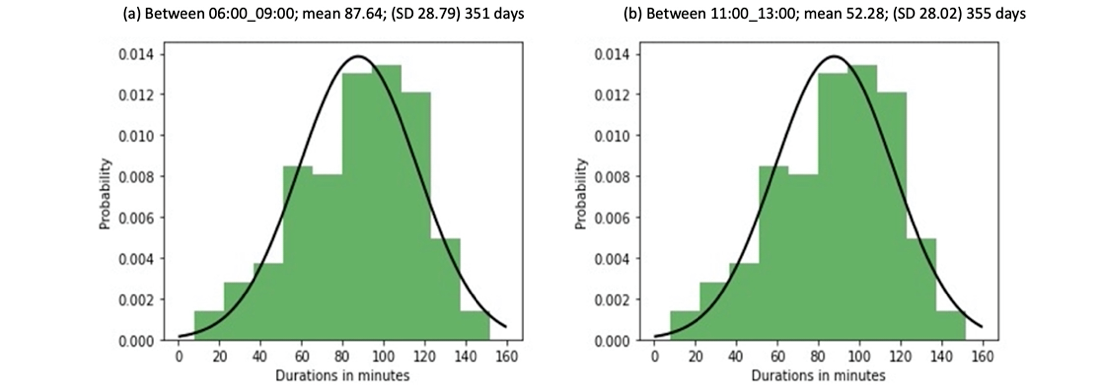

Supplement: Multimedia Appendix 1 [file aging_v7i1e58394_app1.png]
